# ErZhiTianGui Decoction alleviates age-related ovarian aging by regulating mitochondrial homeostasis and inhibiting ferroptosis

**DOI:** 10.1186/s13048-023-01341-9

**Published:** 2024-01-10

**Authors:** Jia Zhicheng, Li Yongqian, Wang Peixuan, Yang Kai, Shi Mengyu, Chen Wen, Liang Qihui, Guo Ying

**Affiliations:** 1 The First Clinical College, Shandong University of Traditional Chinese Medicine, Jinan, China; 2 College of Traditional Chinese Medicine, Shandong University of Traditional Chinese Medicine, Jinan, China; 3 Reproductive and Genetic Center of Integrative Medicine, Affiliated Hospital of Shandong University of Traditional Chinese Medicine, Jinan, China

**Keywords:** ErZhiTianGui Decoction, Ovarian aging, Mitochondrion, Mitophagy, Ferroptosis

## Abstract

**Aim:**

This study was designed to investigate the pharmacological effects and mechanisms of ErZhiTianGui Decoction (EZTG) for age-related ovarian aging in mice.

**Methods:**

This study used naturally aging mice as a model, and EZTG was used for intragastric administration. Ovarian pathological changes, as well as follicular reserve were assessed by hematoxylin and eosin staining, and serum hormone levels (anti-mullerian hormone, follicle-stimulating hormone), mitochondrial reactive oxygen species (ROS) and mitochondrial DNA (mtDNA) damage marker 8-hydroxy-2′-deoxyguanosine(8-OHdG), and lipid peroxidation markers glutathione(GSH) and malondialdehyde(MDA) were determined by enzyme-linked immunosorbent assay. Mitochondrial membrane potential (MMP) levels in ovaries were determined using flow cytometry. The levels of PINK1 and Parkin were observed using immunofluorescence staining. Mitochondrial-derived vesicles (MDVs) and mitochondrial morphology were observed using electron microscopy. Prussian blue staining was used to observe iron ion aggregation in ovarian tissue. The Iron assay kits detected total iron levels. Western blot was used to detect the expression of proteins related to mitochondrial and ferroptosis related genes.

**Results:**

After EZTG treatment, aged mice showed increased ovarian reserve, improved serum hormone levels, increased MMP, GSH levels, and decreased mitochondrial ROS, 8-OHdG, and MDA levels. Immunofluorescence staining showed decreased levels of PINK1 and Parkin, and the same trend was observed for the Western blot. Meanwhile, electron microscopy showed that EZTG improved the mitochondrial morphology in the ovaries of aged mice. EZTG also decreased the total iron and protein levels of Acyl-CoA synthetase long-chain family4 (ACSL4) and increased the protein level of glutathione peroxidase 4 (GPX4) in the ovaries of aged mice.

**Conclusions:**

EZTG can maintain PINK1/Parkin-mediated mitochondrial homeostasis, reduce the lipid peroxidation caused by the accumulation of ROS, and inhibit the occurrence of ferroptosis and delaying ovarian aging. These findings suggest that EZTG may be a promising drug for treating age-related ovarian aging in females.

## Introduction

Age alone has an effect on fertility, women older than 35 are traditionally defined as women with advanced maternal age(AMA) [1], whose fertility declines significantly after the age of 35, with the most significant decrease in ovarian reserve. With the extension of life expectancy and the postponement of fertility intentions worldwide, the proportion of AMA has increased substantially [2, 3]. The mechanism of age-related ovarian dysfunction is not yet clear, and it is currently mainly related to factors such as mitochondrial dysfunction, free radicals and antioxidant systems, telomere and telomerase changes, and cell apoptosis [4]. Exploring the pathogenesis and potential treatment strategies of age-related ovarian aging is currently one of the hotspots in obstetrics and gynecology.

Mitochondria play a crucial role in ovarian aging and reproductive longevity. It has been clarified that mitochondrial dysfunction leads to ovarian aging [5]. Mitochondria are the main source of free radicals and ROS in cells and play a central role in oxidative phosphorylation and redox. The mitochondrial dysfunction produces excessive ROS, and a considerable accumulation of ROS leads to oxidative stress, which in turn affects mitochondrial homeostasis and forms a vicious cycle [6, 7]. Meanwhile, Mitochondria regulate various forms of cell death and play a crucial role in the regulation of ferroptosis [8, 9]. Ferroptosis, an iron-dependent form of non-apoptotic cell death in 2012, has seen exponential growth in research over the past few years [10]. Ferroptosis has also made some research progress in female reproduction, including its role in ovarian cancer [11], endometriosis [12], and polycystic ovary syndrome [13]. However, ferroptosis in age-related ovarian aging has not been elucidated. Regulation of mitochondrial homeostasis may alleviate the occurrence of ferroptosis, thereby alleviating ovarian aging.

Herbal medicine has been used for thousands of years to treat infertility, and they have shown good clinical effects in age-related ovarian aging [14]. Traditional Chinese medicine believes that the kidney is the root of reproduction, and deficiency of the kidney energy is the fundamental cause of age-related ovarian aging. EZTG Decoction, composed of ten herbal ingredients, have the effect of nourishing kidney-qi and tonifying kidney-yin [15]. EZTG has been used in clinical practice for over 20 years with good clinical effects in alleviating ovarian aging and improving pregnancy outcomes for elderly women [16]. To date, no study has been reported on the role of EZTG in alleviating ovarian aging. Therefore, this study used naturally aging mice as a model and administered EZTG intragastrically to explore the role of EZTG in alleviating ovarian aging.

## Methods and material

### Ethical statemen**t**

All animal experiments were conducted in strict accordance with the Guide to the Management and Use of Laboratory Animals issued by the National Institutes of Health. Animal Ethics Committee of Affiliated Hospital of Shandong University of Traditional Chinese Medicine approved all the experimental procedures used in this study (Approval number: 20,220,316).

### Animal models and treatment

Sixty C57/BL6 female mice aged 36 weeks were obtained from JinanPengyYue-laboratory animal breeding Co., Ltd. (SYXK20180015, Jinan, China). All mice were housed in standard conditions with a 12-h light/dark cycle at 22 ± 2 °C and a humidity of 55 ± 5% with free access to food and water. We calculated the dose of EZTG to be taken by mice in the ratio of 9:1 According to the medication instructions, the therapeutic dose of EZTG for a woman weighing 60 kg is 18 g/day. The 60 mice aged 36 weeks were randomly distributed into three groups: an aged control group (old group), a low-dose EZTG group (EZTG-L, 2.7 g/kg/day by gavage), and a high-dose EZTG group (EZTG-H, 8.1 g/kg/day by gavage). The 20 mice aged 8 weeks were used as a young control group. The EZTG Decoction are produced by the Drug Manufacturing Unit of the Affiliated Hospital of Shandong University of Traditional Chinese Medicine, batch number 01-FZ032-03. The composition traditional Chinese herbs of EZTG Decoction include: Cuscuta chinensis (Tu Si Zi), Ligustrum lucidum (Nv Zhen Zi), Herba Ecliptae (Mo Han Lian), Lycium chinense Mill (Gou Qi Zi), Angelica sinensis (Dang Gui), Radix Rehmanniae Preparata (Shu Di Huang), Ligusticum wallichii (Chuan Xiong), Paeonia lactiflora Pall (Bai Shao), Rhizoma cyperi (Xiang Fu), Radix Glycyrrhizae Preparata (Zhi Gan Cao). The EZTG group was gavaged for 12 weeks, while the control group was gavaged with equivalent doses of normal saline. All mice were anesthetized and euthanized, and their blood and ovaries were collected for subsequent use, and the weight of mice and wet weight of ovaries were measured (ovarian index = ovarian mass/body weight, mg/g).

### Histopathology

Ovaries were harvested and fixed with paraformaldehyde at a concentration of 4% for more than 24 h, and dehydration, wax leaching, and embedding were performed. Ovaries were cut into 4 μm sections., which were dewaxed with ethanol and stained with Haematoxylin and eosin dyes. The number of Primordial follicles, Primary follicles, Secondary follicles, and Mature follicles is recorded based on morphological characteristics.

### Serum hormone levels

Serum samples obtained before the mice were euthanized were centrifuged at room temperature for 20 min at 3000×g. Serum hormone levels, including anti-Müllerian hormone (AMH), and follicle-stimulating hormone(FSH), were measured using ELISA kits (F9402-A, F2555-A, Shanghai Fanke Industrial Co., Ltd, Shanghai, China), according to the manufacturer’s instructions.

### Mitochondrial membrane potential flow cytometric assay

Ovarian tissue was cut into small pieces, digesting it completely with trypsin, adding complete culture medium, and collecting the cell suspension. MMP levels were detected using the JC-1 assay kit (E-CK-A301, Elabscience). The cell suspension were removed in each group, and 1 mL JC-1 working solution was added after washing with PBS. Negative and positive control groups were set according to the instructions. The cell suspension stained with JC-1 staining buffer and detected by flow cytometry.

### Mitochondrial ROS assay

ROS assay kits (AO036-100T/96S, Yishijiu Biotechnology Co., Ltd, Jiangsu, China) were utilised to quantify ROS levels in ovary tissue, according to the manufacturer’s instructions. In short, extract mitochondria from ovarian tissue to prepare homogenate. Upon centrifugation, 50 μl Mitochondrial homogenate was mixed with 50 μl catalyst solution and 100 μl(DCFH) solution for 30 min. The 2′,7′-dichlorodihydrofluorescein (DCF) levels were quantified using fluorescence spectrometry with excitation/emission (Ex/Em) at 480/530 nm.

### Enzyme-linked immunosorbent assay

In short, weigh 0.1 g of ovarian tissue, add cold phosphate buffered saline at a ratio of 1:9, and prepare 10% ovarian tissue homogenate, centrifuged at 14,000 × g at 4 °C for 5 min, and collected supernatants. MDA, GSH,8-OHdG was determined by a colorimetric assay kit (F2221-A, F30753-A, F2658-A, Shanghai Fanke Industrial Co., Ltd, Shanghai, China) according to the manufacturer’s guidelines.

### Transmission electron microscope

The ovaries were collected and fixed in Transmission Electron Microscope (TEM) (Servicebio, China) at 4 ℃, and dehydrated with gradient concentrations of ethanol. Then, the ovaries were penetrated and embedded with resin and sliced. Then, the ovary section was stained with uranium acetate and lead citrate. Finally, the ovary section was observed under TEM (Hitachi, Japan), and images were captured.

### Immunofluorescence staining

In brief, Paraformaldehyde-fixed ovaries were dehydrated, embedded, and sliced. Primary antibodies against PINK1(proteintech,23274-1-AP,1: 200) and Parkin(proteintech,66674-1-Ig,1:50) were added at 4 °C overnight, while the secondary antibodies were added to the sample at 37 °C for 30 min (Parkin:Servicebio,GB22301,1:100; Pink1:Servicebio,GB21303,1:200). DAPI was added at room temperature for 10 min. Images were collected by section using a fluorescence microscope camera system and analyzed by Image J software, randomly select areas of the same size from each slice to measure fluorescence intensity, with three replicates per group.

### Prussian blue staining

Prussian blue staining kit (Solarbio, China) was used to detect the deposition of iron-containing heme in ovarian tissue. The paraffin sections were prepared with a thickness of 4 μ m, subjected to routine dehydration and transparency, and then stained with Perls staining solution for 30 min. The slices were thoroughly washed with distilled water for 30–50 s, and the background was lightly stained with eosin staining solution for 15–30 s, followed by rinsing with distilled water for 2–3 s. Finally, the slices were dehydrated with anhydrous ethanol, transparent with xylene, sealed with neutral glue, and placed under a light microscope for observation.

### Iron assay

In short, ovarian tissue was weighed and homogenized in 1 ml ice-cold phosphate-buffered saline, centrifuged at 14,000 × g at 4 °C for 5 min, and collected supernatants. The total iron content in the ovary were detected by the Total Iron Colorimetric Assay Kit (Elabscience, E-BC-K772-M). Spectrophotometry was finally adopted to detect the absorbance at a wavelength of 593 nm.

### Western blot analysis

The expression levels of mitophagy-related proteins PINK1, Parkin and ferroptosis-related proteins GPX4 and ACSL4 were detected by Western blot. After adding radioimmunoprecipitation assay lysis buffer (Beyotime), tissues were ground in a homogenizer and then lysed on ice for 1 h. The Protein concentration was determined using the BCA kit (Solarbio, Beijing, China). The protein loading buffer was added to each sample which was then boiled for 10 min, centrifuged at 4 °C for 10 min and stored. Following protein gel electrophoresis, membrane transfer for 1 h at a constant current of 250 mA in a 4 ℃ transfer solution, blocking, and incubation of primary antibody and secondary antibody, the photographic developer was added to the membrane and the band was developed in ECL chemiluminescence instrument with glyceraldehyde-3-phosphate dehydrogenase as internal reference. The protein bands were analyzed by Image J software (version: v1.8.0). The antibodies as follows: anti-GPX4(Abcam; ab125066; 1:5000), anti-ACSL4(Abcam; ab155282; 1:10000), anti-Parkin(Abcam; ab77924; 1:1000), anti-PINK1(Abcam; ab300623; 1:1000), anti-GAPDH(Proteintech; 60004-1-Ig; 1:5000).

### Statistical analysis

Continuous variables are expressed as mean ± SD throughout this study. Differences between the two groups were analyzed using one-way ANOVA followed by Duncan’s post hoc test. All statistical analyses were performed with the SPSS 25.0 and Image-J statistical software. A *P*-value < 0.05 was considered statistically significant.

## Results

### EZTG improves ovarian function in aged mice

**Fig. 1:**
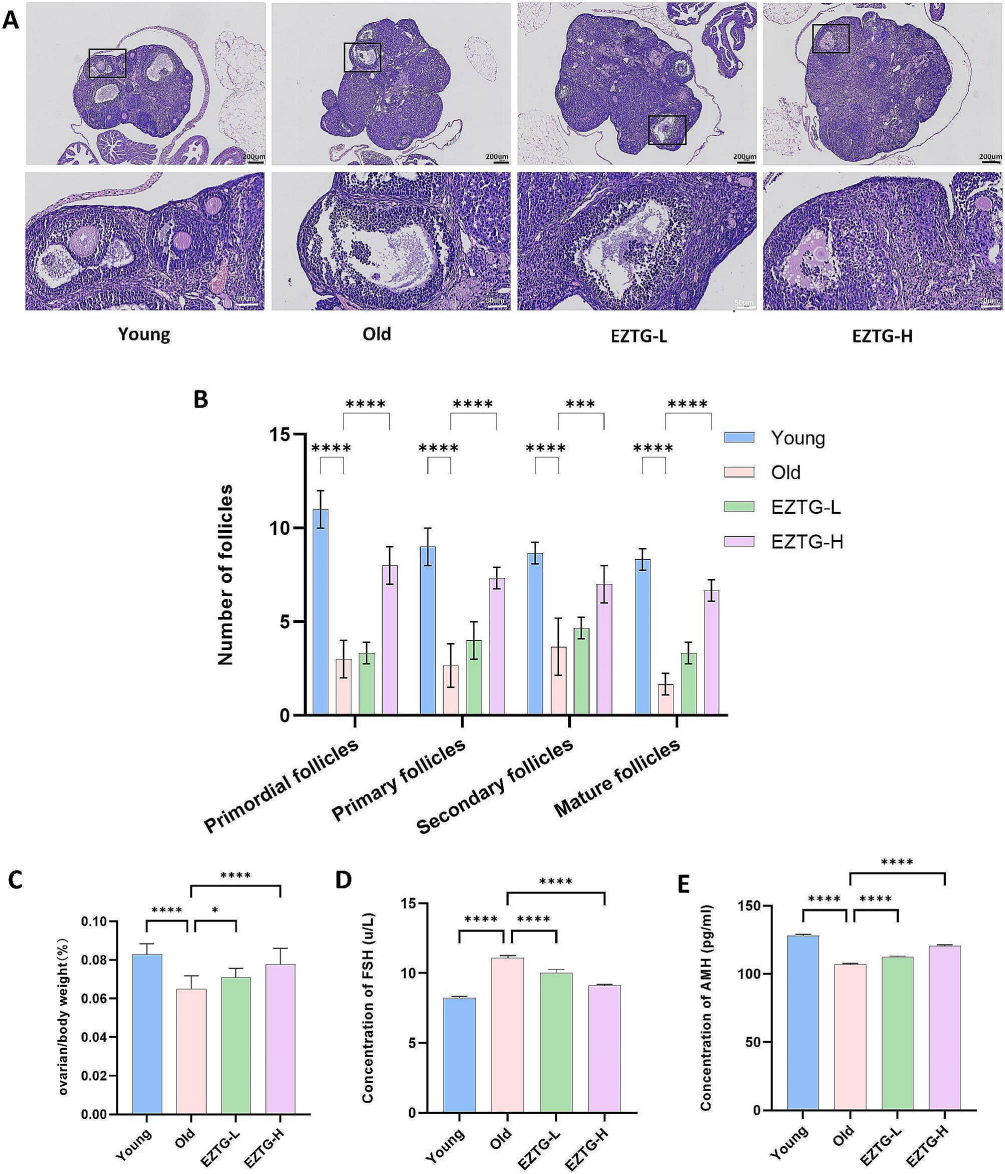
EZTG improves ovarian function in aged mice. (**A**) Ovarian tissue sections with H&E staining in different groups. (**B**) Follicle count at all grades. (**C**) ovarian index (ovarian/body weight). (**D**,**E**) The level of serum follicle stimulating hormone anti-Müllerian hormone. *P < 0.05, **P < 0.01, ***P < 0.001, ****P < 0.0001, All data are expressed as the mean ± SD and at least three independent experiments were performed

The ovaries of the young group are well-developed, and follicles at all grades are visible. Compared with the young group, the old group showed a decrease in the number of corpus luteum in the ovaries, a loose arrangement of granulosa cells, and a decrease in the ovarian index (ovarian/body weight) (Fig. 1A, C). According to the follicle count results, the primordial, primary, secondary, and mature follicles in the EZTG-H group were significantly higher than those in the old group (*P* < 0.05) (Fig. 1B). Compared with the young group, the serum AMH levels in the old group were significantly reduced (*P* < 0.01), while FSH levels were increased. After intervention with medium and high doses of EZTG, compared with the old group, the levels of AMH significantly increased (*P* < 0.01), while the levels of FSH decreased (*P* < 0.05) (Fig. 1D-E).

### EZTG improves mitochondrial function in the ovaries of aged mice

**Fig. 2:**
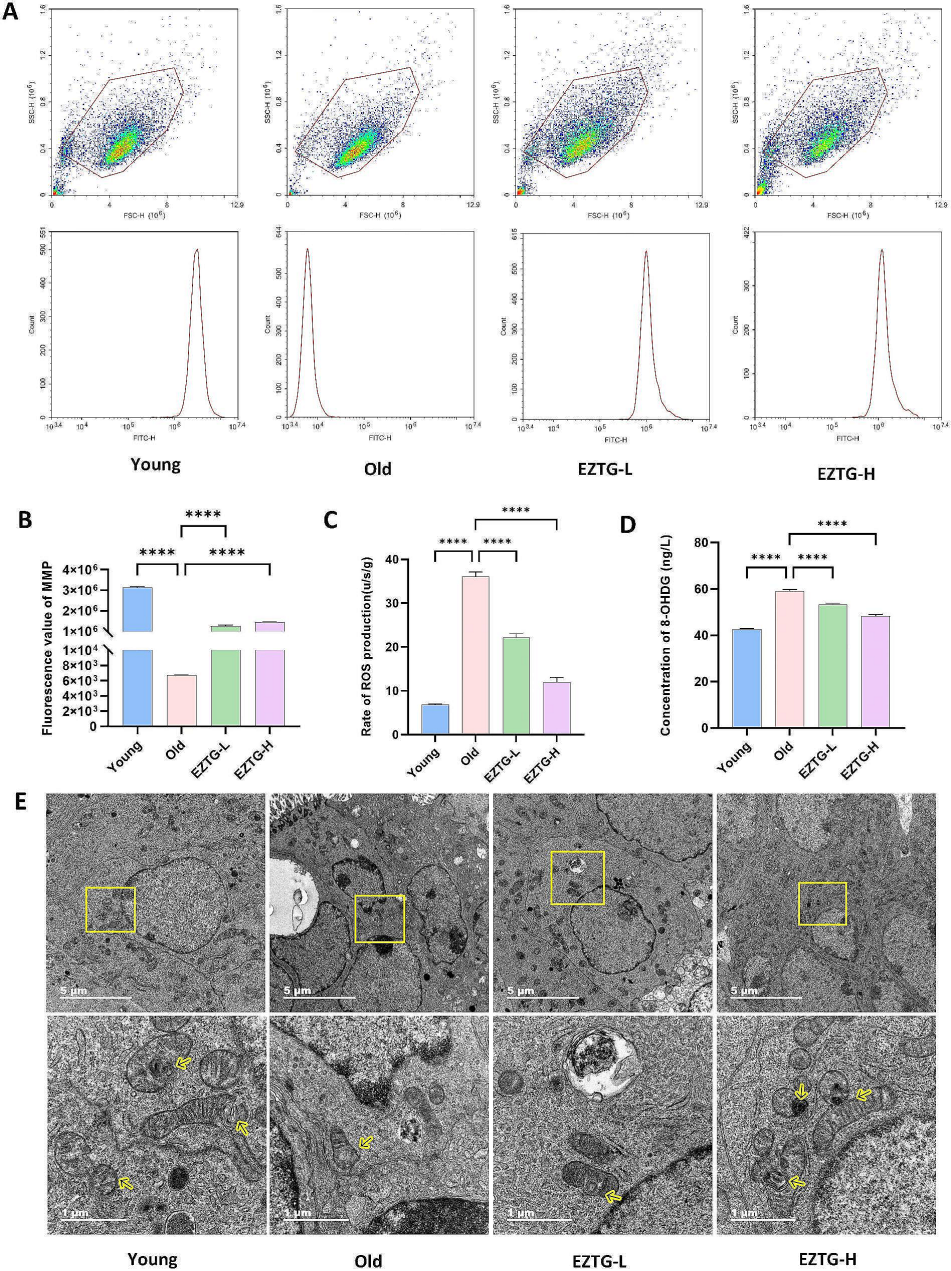
EZTG improves mitochondrial function in the ovaries of aged mice. (**A**,**B**) Flow cytometric analysis of MMPs degradation. (**C**) Rate of Mitochondrial ROS production (**D**) concentration of 8-OHdG. (**E**) Observation of mitochondrial ultrastructure and MDVs in ovaries under transmission electron microscopy, the yellow arrow identifies MDVs, scale bar + = 5 μm. *P < 0.05, **P < 0.01, ***P < 0.001, ****P < 0.0001, All data are expressed as the mean ± SD and at least three independent experiments were performed

In order to explore the effect of EZTG on mitochondrial dysfunction, we used JC-1 and flow cytometry to study the level of MMP in the ovary. Compared with the young group, the MMP in the old group was significantly lower. However, in EZTG-L and EZTG-H groups, the MMP increased significantly compared with the old group (*P* < 0.05) (Fig. 2A.B). At the same time, we measured the levels of markers of mitochondrial oxidative damage 8-OHdG and mitochondrial ROS. Compared with the young group, the 8-OHdG and mitochondrial ROS in the old group were significantly increased. In the EZTG-L and EZTG-H groups, 8-OHdG and mitochondrial ROS were significantly decreased compared to the old group (*P* < 0.05)(Fig. 2C, D). The mitochondrial ultrastructure of ovarian tissue was observed using TEM. In the young group, mitochondrial morphology is intact, with more MDVs. The number of mitochondria in the old group decreased, with fragmented morphology and less MDVs. However, the administration of EZTG maintained mitochondrial homeostasis (Fig. 2E).

### EZTG improves the level of mitophagy in the ovaries of aged mice

**Fig. 3:**
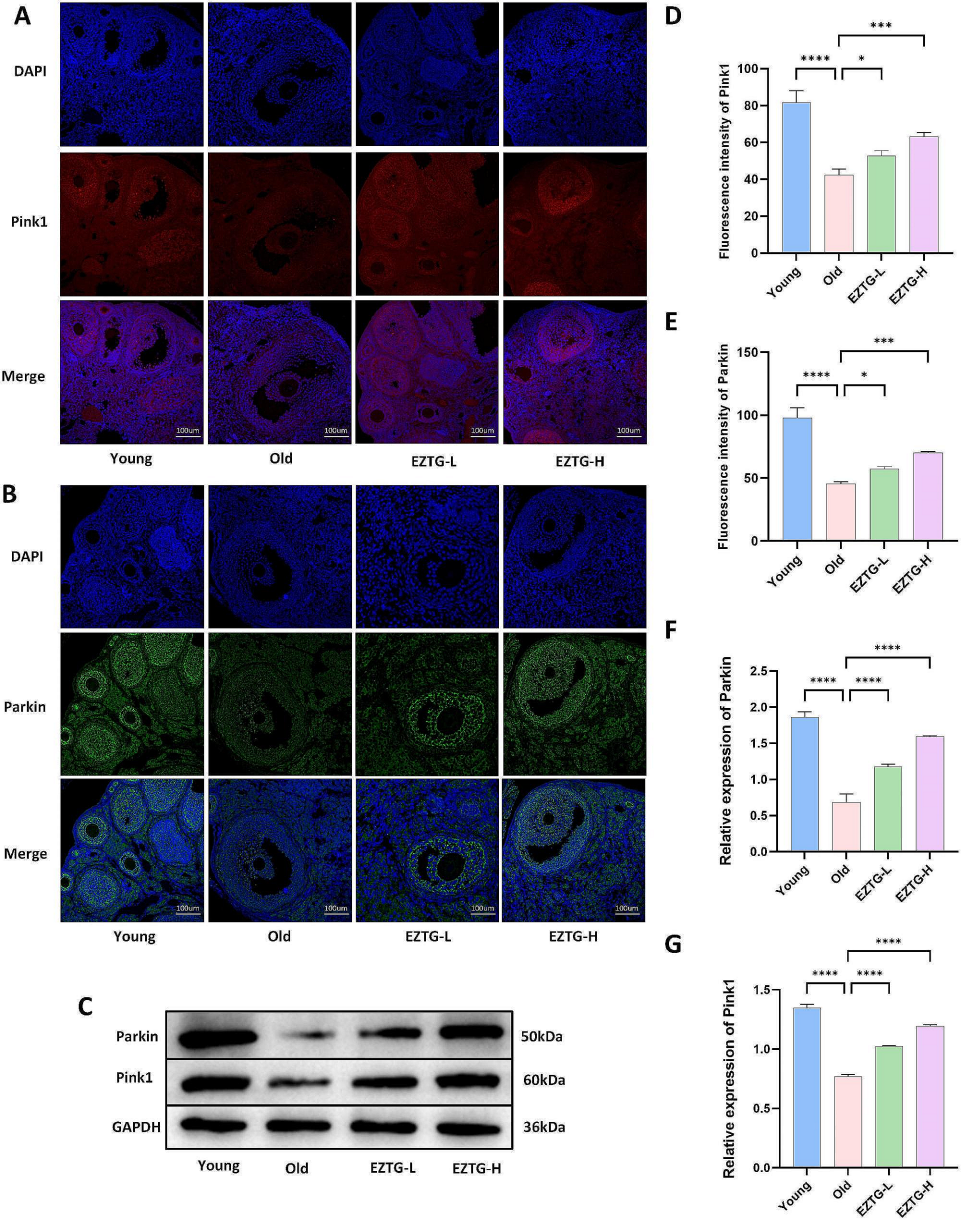
EZTG improves the level of mitophagy in the ovaries of aged mice. (**A**,**B**) Immunofluorescence images of the PINK1 and Parkin. (**C**) Representative protein bands of PINK1 and Parkin. (**D**,**E**) Quantification of Immunofluorescence staining for PINK1 and Parkin. (**F**,**G**) The corresponding quantitative statistical results of PINK1 and Parkin protein. *P < 0.05, **P < 0.01, ***P < 0.001, ****P < 0.0001, All data are expressed as the mean ± SD and at least three independent experiments were performed

To detect mitophagy levels, we used immunofluorescence staining to measure the expression of Parkin and PINK1 in the ovaries and measured protein expression using Western blot. Compared with the young group, the fluorescence expression levels of PINK and Parkin in the old group were significantly reduced. The fluorescence expression levels in the EZTG-L and EZTG-H groups were significantly higher than in the old group (Fig. 3A, B, D, E). Similarly, the results of Western blot also showed a similar trend (Fig. 3C, F, G).

### EZTG can alleviate ferroptosis in the ovaries of aged mice

**Fig. 4:**
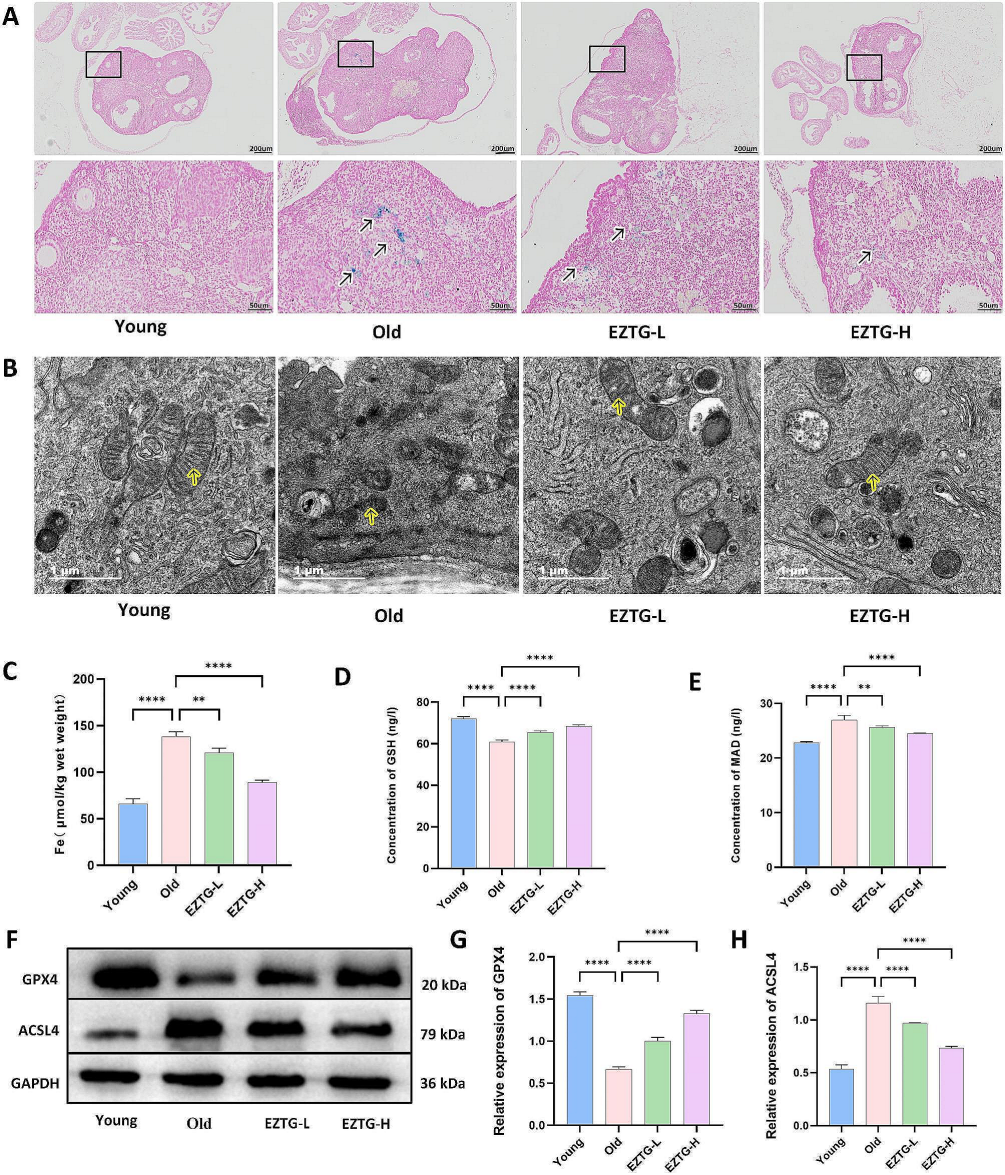
EZTG can alleviate ferroptosis in the ovaries of aged mice. (**A**) Ovarian tissue sections with Prussian blue staining, scale bar + = 50 μm. (**B**) Observation of Mitochondrial morphology in ovaries under transmission electron microscopy, the yellow arrow identifies mitochondrial cristae, scale bar + = 1 μm. (**C**.**D**.**E**) Quantitative analysis of iron ions, GSH and MDA in ovaries. (**F**) Representative protein bands of GPX4 and ACSL4. (**G**.**H**) The corresponding quantitative statistical results of GPX4 and ACSL4 protein. *P < 0.05, **P < 0.01, ***P < 0.001, ****P < 0.0001, All data are expressed as the mean ± SD and at least three independent experiments were performed

Prussian blue staining was used to evaluate iron deposition in ovarian tissue. The results showed a significant increase in iron deposition in the old group compared to the young group. The iron deposition in the EZTG-L and EZTG-H groups was significantly reduced compared to the old group (Fig. 4A). Similarly, there is a similar trend in the levels of Iron ions in the ovaries (Fig. 4C). TEM observed the mitochondrial morphological changes showed that the mitochondria in the old group were smaller than those in the young group, the mitochondrial membrane density was condensed, mitochondria crista was reduced, and the outer mitochondrial membrane was ruptured. The mitochondrial morphology of the ovaries in both EZTG-L and EZTG-H groups improved (Fig. 4B). Lipid peroxidation is one of the main mechanisms of Ferroptosis. We evaluated the levels of GSH and MDA in ovarian tissue. Compared to the young group, the GSH level in the old group decreased while the MDA level increased. The GSH levels in the EZTG-L and EZTG-H groups increased, while MDA levels decreased compared to the old group (Fig. 4D, E). The protein blotting results showed a decrease in GPX4 levels in the old group, while an increase in ACSL4 levels was observed compared to the young group. The levels of GPX4 increased in the EZTG-L and EZTG-H groups, while the levels of ACSL4 decreased compared to the old group (Fig. 4F, G, H).

## Discussion

Ovarian aging in old women is the main cause of infertility and an intractable clinical issue. Whether using the customized IVF-ET regimen [17] or the combination of other drugs, such as Dehydroepiandrosterone, Luteinizing hormone [18], improving the clinical pregnancy outcome of AMA is challenging. This study established a mouse model of natural aging to study the effect of EZTG on alleviating age-related ovarian aging. The experimental results indicate that EZTG can improve ovarian endocrine function and increase the number of follicles. In addition, EZTG can maintain PINK1/Parkin-mediated mitochondrial homeostasis, reduce the lipid peroxidation caused by the accumulation of ROS, and inhibit the occurrence of ferroptosis and delaying ovarian aging.

Studies based on clinical samples reveal that Erzhi-Tiangui Granule alleviates ovarian aging and its effect on follicular fluid and granulosa cells in AMA. EZTG can improve embryo quality in elderly infertile women with kidney Qi and Yin deficiency syndromes and reduce the apoptotic effect in the follicular fluid [19]. Meanwhile, EZTG increased oocyte fertilisation rate and affected granulosa cells’ mRNA expression profile in patients with premature ovarian failure [20]. In this study, we observed that aged mice showed increased ovarian reserve, improved serum hormone levels after EZTG treatment. Many famous traditional Chinese medicines in EZTG include Lycium chinense Mill, Angelica sinensis, Paeonia lactiflora Pall, etc. Lycium chinense Mill can repairs ovarian injuries by scavenging oxidative products 8-hydroxy-2’ -deoxyguanosine and increasing anti-mullerian hormone expression in mice with ovarian injuries induced by repeated superovulation [21]. Angelica sinensis, also known as “female ginseng”, is renowned for treating various gynaecological conditions [22]. Angelica sinensis extract could enhance the invasion and migration ability of follicle micro-vascular endothelial-like cells of chicken in vitro and promote the proliferation of granulosa cells [23]. Paeonia lactiflora Pall has long been used to treat female infertility and recurrent miscarriages. The water-extract Paeonia lactiflora Pall increases ovarian expression of angiogenic factors and follicular development regulatory factors in aged female mice [24].

Mitochondrial dysfunction induces ovarian aging, not only perturbs oocyte-cumulus crosstalk in the ovary [5] but also affects the processes during oocyte meiosis and the developmental potential of embryos [25]. Mitochondrial dysfunction is reflected in many aspects, including the decrease of MMP, the increase of mtDNA mutations, and the accumulation of ROS, etc [26, 27]. In our study, we observed the decrease of MMP, the increase of ROS and 8-OHdG in the ovary of aging mice, indicating mitochondrial dysfunction. After further administration of EZTG, mitochondrial dysfunction was alleviated at these levels. The decrease of MMP, considered a sign of mitochondrial dysfunction, is typically associated with increased production of ROS [28, 29]. Mitochondria are the main source of free radicals and ROS in cells [30]. The mitochondrial disorder produces excessive ROS, and a considerable accumulation of ROS leads to oxidative stress, which in turn affects mitochondrial homeostasis and forms a vicious cycle [6, 7]. In elderly women receiving assisted reproductive therapy, the concentration of ROS in their oocytes, cumulus oophorus and follicular fluid increase [31], while antioxidant decreases [32]. The mtDNA is vulnerable to ROS attack due to lack of histone protection and overlaps with ROS production sites in the Inner mitochondrial membrane [33], leading to mitochondrial dysfunction and premature aging [34]. Among many types of mtDNA oxidative lesions, 8-OHdG is an indicator of steady-state level of oxidative damage occurring within mtDNA [35].

Mitophagy is a critical mitochondrial quality control mechanism that maintains mitochondrial homeostasis by selectively eliminating damaged or excessive mitochondria [36]. Mitophagy activity decreases with age, and its upregulation may improve symptoms of age-related phenotypes [37]. Our study found that the levels of PINK1 and Parkin in the ovaries of aged mice decreased, indicating a decrease in mitophagy levels. At the same time, we observed changes in MDVs in the ovaries of aged mice under electron microscopy, EZTG reversed this impact to some extent. PINK1/Parkin mainly regulates ubiquitin-dependent mitophagy, which is one of the main mechanisms of autophagy and is related to its initiation [38]. Knockout of PINK1 in Drosophila leads to infertility and mitochondrial dysfunction, which can be alleviated by overexpression of Parkin [39]. In addition to mitophagy, Parkin and PINK1 have been shown to control mitochondrial quality through a mitophagy-independent mechanism of mitochondria turnover via the formation of MDVs [40].

Mitochondria regulate various forms of cell death and play a crucial role in the regulation of ferroptosis [8, 9]. The morphological changes of mitochondria, including reduced mitochondrial volume, increased bilayer membrane density and reduction or disappearance of mitochondrial cristae, are also one of the signs of ferroptosis. Ferroptosis, a novel form of regulated cell death driven by iron-dependent lipid peroxidation, is characterized by the increase of free iron and accumulated lipid peroxides, which produces intracellular ROS [41]. Iron ion, a major indicator of ferroptosis, oxidizes lipidsin a Fenton-like manner, thus promoting ferroptosis [42]. Depleting intracellular GSH leads to the decrease of GPX4 activity, leading to the disorder of lipid oxide metabolism, thus promoting ferroptosis. Among them, MDA is one of the products of Lipid peroxidation [43]. GPX4, at the crossroads of lipid homeostasis and ferroptosis, the functional inactivation of GPX4 results in an increased lipid-ROS accumulation and subsequent lipid peroxidation [44]. Meanwhile, ACSl4 promotes sensitivity to ferroptosis by shaping cellular lipid components [45]. In our study, we observed the aggregation of iron ions, the increase of Lipid peroxidation products, the decrease of GSH and GPX4, and the increase of ACSL4 in the ovaries of aged mice. Similarly, we observed reduced mitochondrial volume, increased bilayer membership density and reduction or disappearance of mitochondrial cristae in the ovaries of aged mice. This indicates that ferroptosis occurs in age-related ovarian aging. EZTG alleviated the occurrence of ferroptosis to varying degrees.

However, our study had some limitations, including a lack of in vitro validation experiments to confirm the relationship between mitochondrial homeostasis and inhibiting ferroptosis in ovarian aging. In addition, due to the disordered estrous cycle of elderly mice, on the day of mouse euthanasia, not all mice were in the same estrous cycle stage, which may lead to deviations in corpus luteum quantity and blood hormone levels. At present, almost all relevant animal experimental studies have yet to mention how to control this variable, which is also an idea that we need to advance continuously.

## Conclusions

**Fig. 5:**
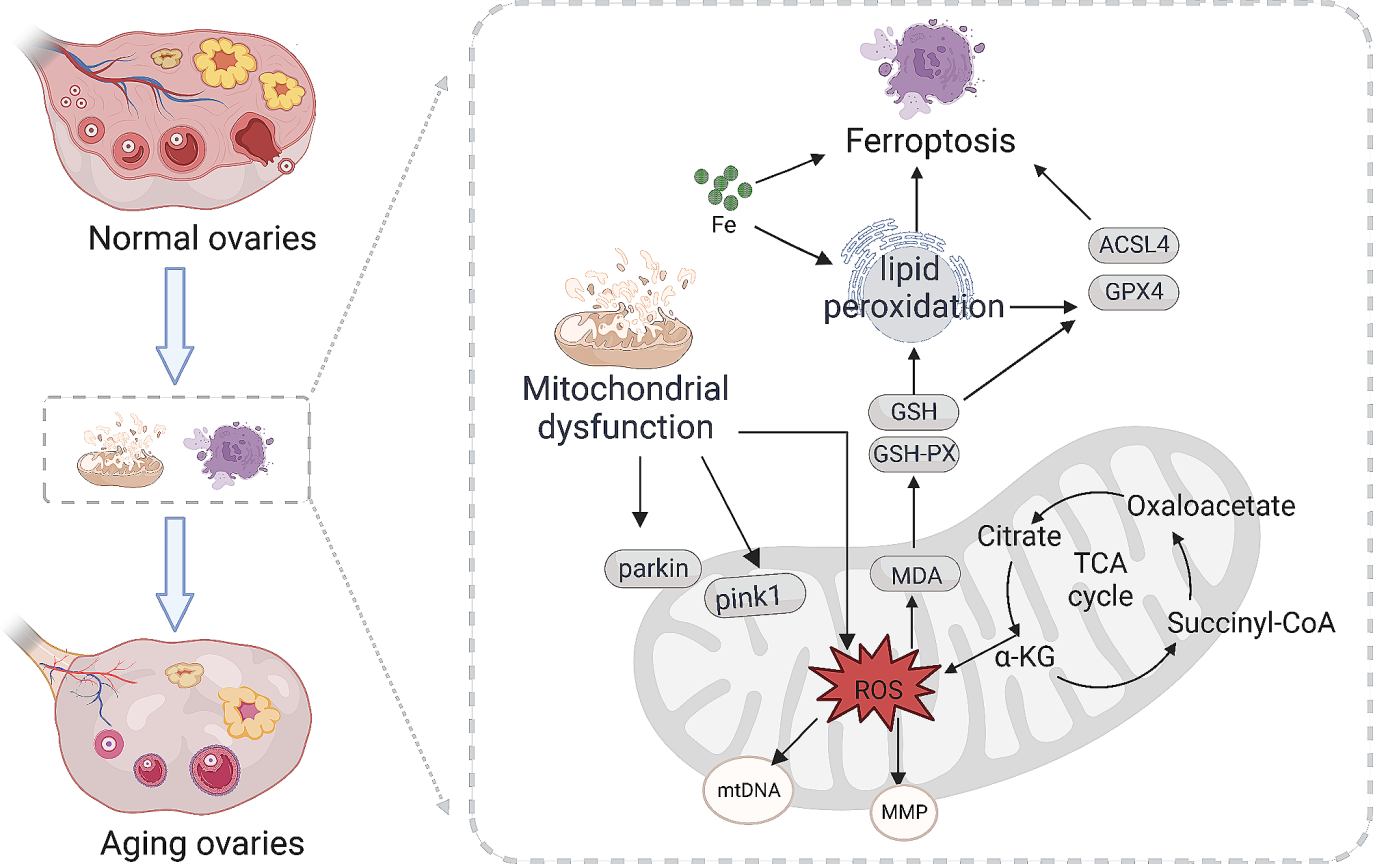
Schematic illustration showing that EZTG alleviates age-related ovarian aging by regulating mitochondrial homeostasis to inhibit ferroptosis

In addition, EZTG can maintain PINK1/Parkin-mediated mitochondrial homeostasis, reduce the lipid peroxidation caused by the accumulation of ROS, and inhibit the occurrence of ferroptosis and delaying ovarian aging (Fig. 5). These findings suggest that EZTG may be a promising drug for treating age-related ovarian aging in females.

## Data Availability

The raw data supporting the conclusions of this article will bemade available by the authors, without undue reservation.

## References

[1] Female age-related fertility decline (2014). Comm Opin No 589 Fertility Steril.

[2] Ben Messaoud K, Bouyer J, de La Rochebrochard E (2020). Infertility treatment in France, 2008–2017: a challenge of growing treatment needs at older ages. Am J Public Health.

[3] Schmidt L, Sobotka T, Bentzen JG, Nyboe Andersen A (2012). Demographic and medical consequences of the postponement of parenthood. Hum Reprod Update.

[4] Zhang J, Chen Q, Du D, Wu T, Wen J, Wu M, Zhang Y, Yan W, Zhou S, Li Y (2019). Can ovarian aging be delayed by pharmacological strategies?. Aging.

[5] Chiang JL, Shukla P, Pagidas K, Ahmed NS, Karri S, Gunn DD, Hurd WW, Singh KK (2020). Mitochondria in Ovarian Aging and Reproductive Longevity. Ageing Res Rev.

[6] Yamada Y, Takano Y, Satrialdi; Abe J, Hibino M, Harashima H. Therapeutic strategies for regulating mitochondrial oxidative stress. Biomolecules. 2020;10. 10.3390/biom10010083 10.3390/biom10010083 PMC7023101 31948035

[7] Huang Z, Chen Y, Zhang Y. Mitochondrial reactive oxygen species cause major oxidative mitochondrial DNA damages and repair pathways. J Biosci. 2020;45. 32661211

[8] Chipuk JE, Mohammed JN, Gelles JD, Chen Y (2021). Mechanistic connections between mitochondrial biology and regulated cell death. Dev Cell.

[9] Gan B. Mitochondrial regulation of ferroptosis. J Cell Biol. 2021;220. 10.1083/jcb.202105043 10.1083/jcb.202105043 PMC8329737 34328510

[10] Chen X, Li J, Kang R, Klionsky DJ, Tang D (2021). Ferroptosis: machinery and regulation. Autophagy.

[11] Zhang C, Liu N. Ferroptosis, necroptosis, and pyroptosis in the occurrence and development of ovarian cancer. Front Immunol. 2022;13. 10.3389/fimmu.2022.920059 10.3389/fimmu.2022.920059 PMC9361070 35958626

[12] Ni Z, Li Y, Song D, Ding J, Mei S, Sun S, Cheng W, Yu J, Zhou L, Kuang Y, et al. Iron-overloaded follicular fluid increases the risk of endometriosis-related infertility by triggering granulosa cell ferroptosis and oocyte dysmaturity. Cell Death Dis. 2022;13. 10.1038/s41419-022-05037-8 10.1038/s41419-022-05037-8 PMC9253011 35787614

[13] Tan W, Dai F, Yang D, Deng Z, Gu R, Zhao X, Cheng Y. MiR-93-5p promotes granulosa cell apoptosis and ferroptosis by the NF-kB signaling pathway in polycystic ovary syndrome. Front Immunol. 2022;13. 10.3389/fimmu.2022.967151 10.3389/fimmu.2022.967151 PMC9626535 36341347

[14] Feng J, Wang J, Zhang Y, Zhang Y, Jia L, Zhang D, Zhang J, Han Y, Luo S. The efficacy of complementary and alternative medicine in the treatment of female infertility. Evidence-based Complement Altern Med. 2021;2021. 10.1155/2021/6634309

[15] Han QS, Zhou Y, Chen W, Song JY, Sun ZG (2023). The role of Erzhi Tiangui formula in expected poor ovarian responders undergoing in vitro fertilization-embryo transfer: a multicenter, randomized, double-blind, placebo-controlled trial. Med (Baltim).

[16] Sun J, Song JY, Dong Y, Xiang S, Guo Q. Erzhi Tiangui granules improve in Vitro Fertilization outcomes in Infertile Women with Advanced Age. Evidence-based Complement Altern Med. 2021;2021. 10.1155/2021/9951491 10.1155/2021/9951491 PMC8378947 34422084

[17] Gleicher N, Kushnir VA, Albertini DF, Barad DH (2016). Improvements in IVF in women of advanced age. J Endocrinol.

[18] Conforti A, Esteves SC, Humaidan P, Longobardi S, D’Hooghe T, Orvieto R, Vaiarelli A, Cimadomo D, Rienzi L, Ubaldi FM, et al. Recombinant human luteinizing hormone co-treatment in ovarian stimulation for assisted reproductive technology in women of advanced reproductive age: a systematic review and meta-analysis of randomized controlled trials. Reproductive Biology and Endocrinology: RB&E. 2021;19(91). 10.1186/s12958-021-00759-4 10.1186/s12958-021-00759-4 PMC8215738 34154604

[19] Sun J, Song JY, Dong Y, Xiang S, Guo Q. Erzhi Tiangui Granules Improve In Vitro Fertilization Outcomes in Infertile Women with Advanced Age. Evidence-based complementary and alternative medicine: eCAM 2021, 2021:9951491. 10.1155/2021/9951491 10.1155/2021/9951491 PMC8378947 34422084

[20] Liu DQ, Wei CF, Zhang X, Xiang S, Lian F (2023). MicroRNA profiling reveals effects of Erzhi Tiangui granules on kidney deficiency diminished ovarian reserve: a randomized trial. Med (Baltim).

[21] Liu B, Wang JL, Wang XM, Zhang C, Dai JG, Huang XM, Gao JM (2020). Reparative effects of lycium barbarum polysaccharide on mouse ovarian injuries induced by repeated superovulation. Theriogenology.

[22] Hook IL (2014). Danggui to Angelica Sinensis root: are potential benefits to European women lost in translation? A review. J Ethnopharmacol.

[23] Chen H, Chen X, Ping Z, Jiang X, Ge M, Ma J, Yu W (2022). Promotion effect of angelica Sinensis extract on angiogenesis of chicken preovulatory follicles in vitro. Poult Sci.

[24] Park MJ, Han SE, Kim HJ, Heo JD, Choi HJ, Ha KT, Yang SW, Lee KS, Kim SC, Kim CW (2020). Paeonia lactiflora improves ovarian function and oocyte quality in aged female mice. Anim Reprod.

[25] Kirillova A, Smitz JEJ, Sukhikh GT, Mazunin I. The role of Mitochondria in Oocyte Maturation. Cells. 2021;10. 10.3390/cells10092484 10.3390/cells10092484 PMC8469615 34572133

[26] Cen X, Zhang M, Zhou M, Ye L, Xia H. Mitophagy regulates neurodegenerative diseases. Cells. 2021;10. 10.3390/cells10081876 10.3390/cells10081876 PMC8392649 34440645

[27] Miwa S, Kashyap S, Chini E, von Zglinicki T. Mitochondrial dysfunction in cell senescence and aging. J Clin Investig. 2022;132. 10.1172/jci158447 10.1172/JCI158447 PMC9246372 35775483

[28] Passos JF, Nelson G, Wang C, Richter T, Simillion C, Proctor CJ, Miwa S, Olijslagers S, Hallinan J, Wipat A, et al. Feedback between p21 and reactive oxygen production is necessary for cell senescence. Mol Syst Biol. 2010;6. 10.1038/msb.2010.5 10.1038/msb.2010.5 PMC2835567 20160708

[29] Park SU, Walsh L, Berkowitz KM (2021). Mechanisms of ovarian aging. Reprod (Cambridge England).

[30] Murphy MP (2009). How mitochondria produce reactive oxygen species. Biochem J.

[31] Ávila J, González-Fernández R, Rotoli D, Hernández J, Palumbo A. Oxidative Stress in Granulosa-Lutein Cells From In Vitro Fertilization Patients. Reproductive sciences (Thousand Oaks, Calif.). 2016;23:1656–1661. 10.1177/1933719116674077 10.1177/1933719116674077 27821562

[32] Shi L, Zhang J, Lai Z, Tian Y, Fang L, Wu M, Xiong J, Qin X, Luo A, Wang S (2016). Long-term moderate oxidative stress decreased Ovarian Reproductive function by reducing follicle quality and progesterone production. PLoS ONE.

[33] Kasapoğlu I, Seli E. Mitochondrial dysfunction and ovarian aging. Endocrinology. 2020;161. 10.1210/endocr/bqaa001 10.1210/endocr/bqaa001 31927571

[34] Kujoth GC, Hiona A, Pugh TD, Someya S, Panzer K, Wohlgemuth SE, Hofer T, Seo AY, Sullivan R, Jobling WA (2005). Mitochondrial DNA mutations, oxidative stress, and apoptosis in mammalian aging. Sci (New York N Y).

[35] Cividini F, Scott BT, Dai A, Han W, Suarez J, Diaz-Juarez J, Diemer T, Casteel DE, Dillmann WH (2016). O-GlcNAcylation of 8-Oxoguanine DNA glycosylase (Ogg1) impairs oxidative mitochondrial DNA lesion repair in Diabetic hearts. J Biol Chem.

[36] Sun N, Youle RJ, Finkel T (2016). The mitochondrial basis of aging. Mol Cell.

[37] Liu L, Liao X, Wu H, Li Y, Zhu Y, Chen Q (2020). Mitophagy and its contribution to Metabolic and Aging-Associated disorders. Antioxid Redox Signal.

[38] Li J, Yang D, Li Z, Zhao M, Wang D, Sun Z, Wen P, Dai Y, Gou F, Ji Y (2023). PINK1/Parkin-mediated mitophagy in neurodegenerative diseases. Ageing Res Rev.

[39] Clark IE, Dodson MW, Jiang C, Cao JH, Huh JR, Seol JH, Yoo SJ, Hay BA, Guo M (2006). Drosophila pink1 is required for mitochondrial function and interacts genetically with parkin. Nature.

[40] McLelland GL, Soubannier V, Chen CX, McBride HM, Fon EA (2014). Parkin and PINK1 function in a vesicular trafficking pathway regulating mitochondrial quality control. EMBO J.

[41] Jiang X, Stockwell BR, Conrad M (2021). Ferroptosis: mechanisms, biology and role in disease. Nat Rev Mol Cell Biol.

[42] Stockwell BR, Jiang X, Gu W (2020). Emerging mechanisms and Disease Relevance of Ferroptosis. Trends Cell Biol.

[43] Gaschler MM, Stockwell BR (2017). Lipid peroxidation in cell death. Biochem Biophys Res Commun.

[44] Forcina GC, Dixon SJ (2019). GPX4 at the crossroads of lipid homeostasis and Ferroptosis. Proteomics.

[45] Doll S, Proneth B, Tyurina YY, Panzilius E, Kobayashi S, Ingold I, Irmler M, Beckers J, Aichler M, Walch A (2017). ACSL4 dictates ferroptosis sensitivity by shaping cellular lipid composition. Nat Chem Biol.

